# Supplementary material for: Comprehensive transcriptomic analysis shows disturbed calcium homeostasis and deregulation of T lymphocyte apoptosis in inclusion body myositis
Source: J Neurol. 2022 Mar 2;269(8):4161–73. doi: 10.1007/s00415-022-11029-7 (PMC9293871; doi:10.1007/s00415-022-11029-7)
Supplement: Supplementary file 1 — Supplementary file1 (PDF 7388 KB) [file 415_2022_11029_MOESM1_ESM.pdf]

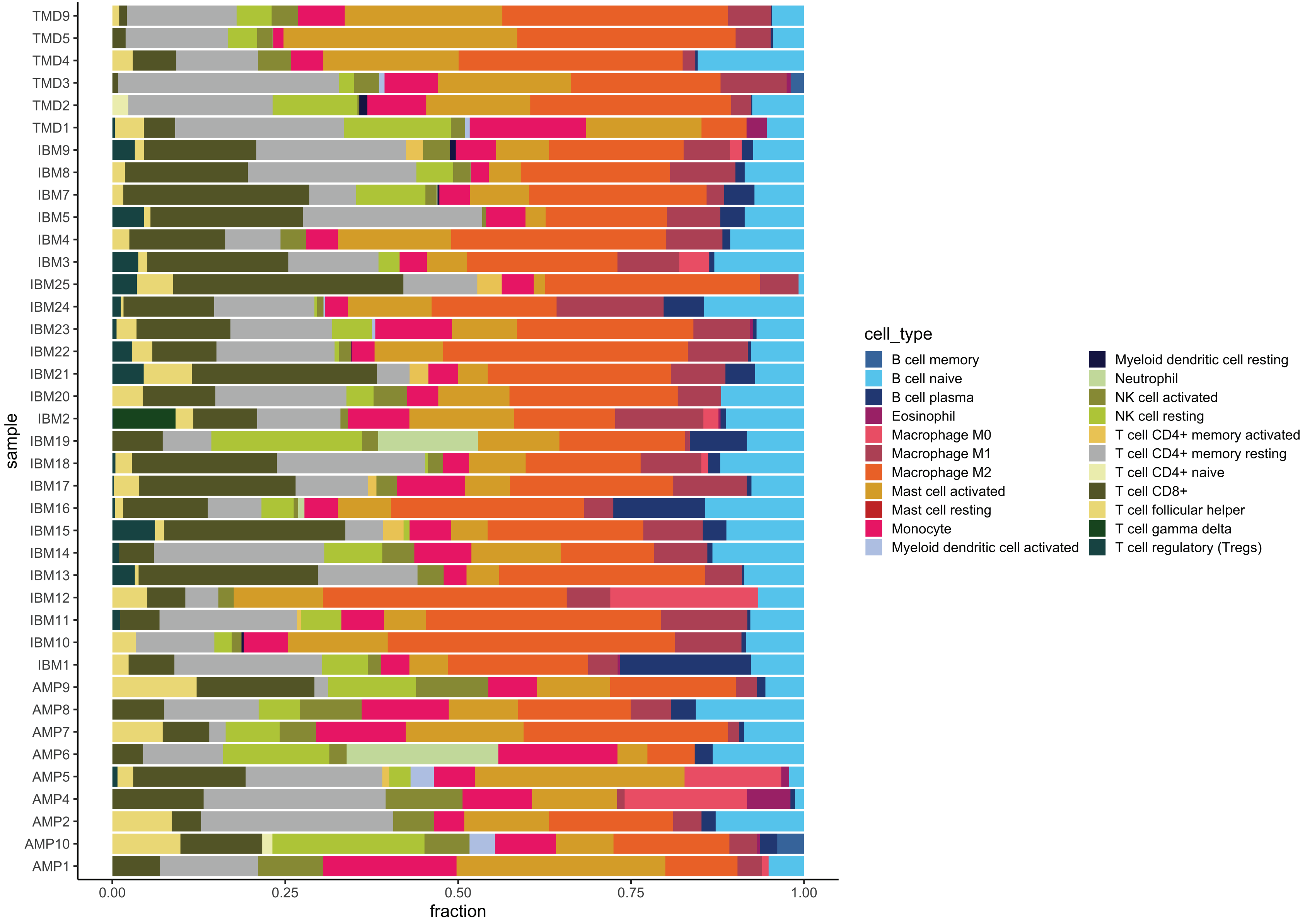

Supplementary Figure 1: Estimation of immune cell fraction

Estimation of immune cell fraction using Immunedeconv and CIBERSORT in individual samples of the three cohorts: IBM, TMD and Amputees. Twenty-two different immune cell types are represented with respective colors as shown in the legend.

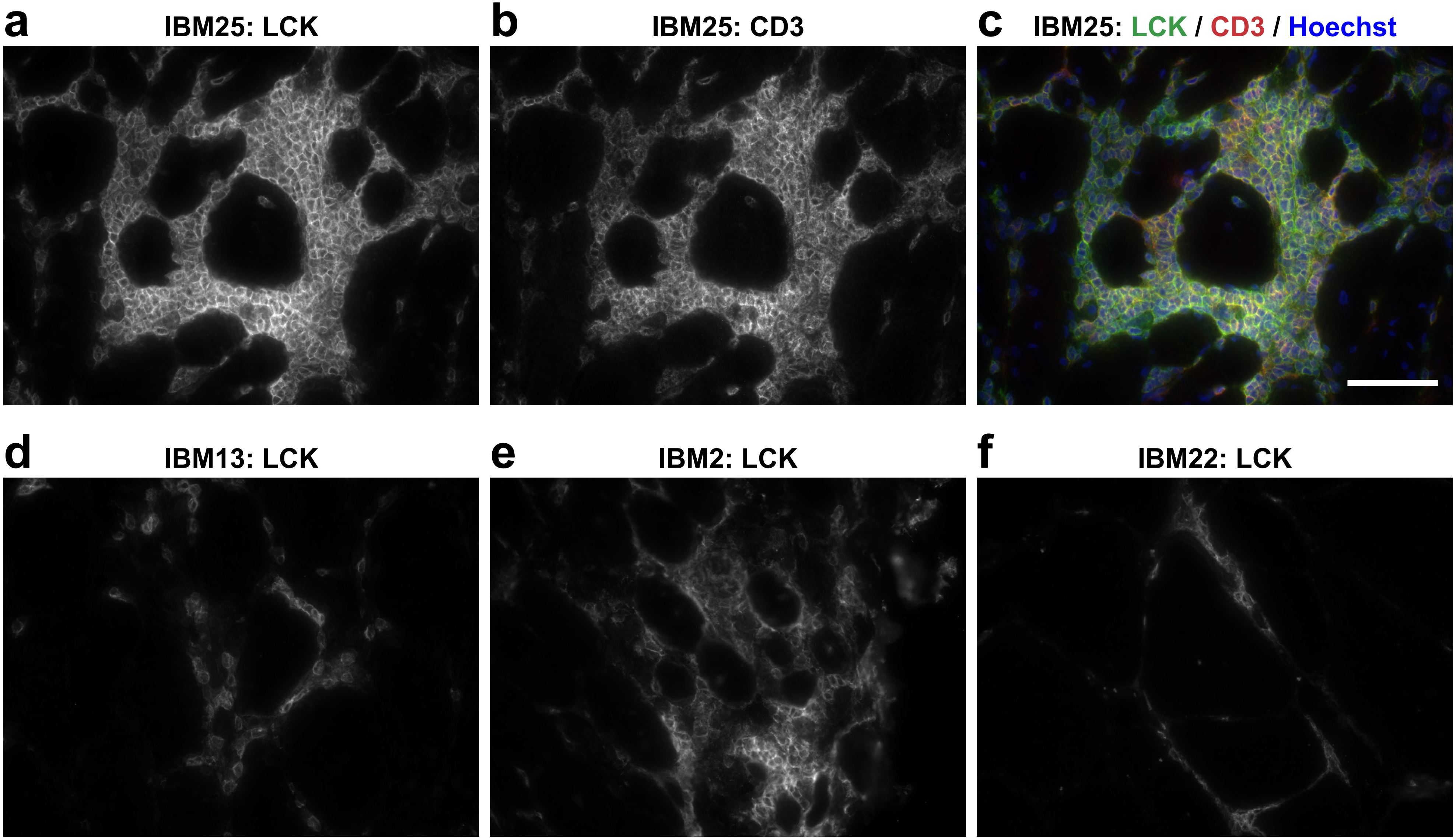

Supplementary Figure 2

a–c Muscle biopsy of IMB25 showing massive inflammatory infiltrate. **(a)** Strong LCK staining is seen in most lymphocytes, which are identified as T-cells by the anti-CD3 antibody **(b)**.The merged image **(c)** shows LCK in green, CD3 in red, Hoechst in blue.
d–e A variable number of T-cells expressing LCK are observed in three additional IBM muscle biopsies showing inflammation.
Scale bar = 50 µm.
